# Chronic Social Defeat Stress Increases Brain Permeability to Ghrelin in Male Mice

**DOI:** 10.1523/ENEURO.0093-24.2024

**Published:** 2024-07-10

**Authors:** Andrea Smith, Brenna MacAulay, Jessica Scheufen, Abagael Hudak, Alfonso Abizaid

**Affiliations:** Department of Neuroscience, Carleton University, Ottawa, Ontario K1S5B6, Canada

**Keywords:** blood–brain barrier, chronic social stress, dopamine, energy balance, food intake, ghrelin, hypothalamus, nucleus accumbens, ventral tegmental area

## Abstract

Ghrelin is a stomach-derived hormone that increases feeding and is elevated in response to chronic psychosocial stressors. The effects of ghrelin on feeding are mediated by the binding of ghrelin to the growth hormone secretagogue receptor (GHSR), a receptor located in hypothalamic and extrahypothalamic regions important for regulating food intake and metabolic rate. The ability of ghrelin to enter the brain, however, seems to be restricted to circumventricular organs like the median eminence and the brainstem area postrema, whereas ghrelin does not readily enter other GHSR-expressing regions like the ventral tegmental area (VTA). Interestingly, social stressors result in increased blood–brain barrier permeability, and this could therefore facilitate the entry of ghrelin into the brain. To investigate this, we exposed mice to social defeat stress for 21 d and then peripherally injected a Cy5-labelled biologically active ghrelin analog. The results demonstrate that chronically stressed mice exhibit higher Cy5-ghrelin fluorescence in several hypothalamic regions in addition to the ARC, including the hippocampus and midbrain. Furthermore, Cy5-ghrelin injections resulted in increased FOS expression in regions associated with the reward system in chronically stressed mice. Further histologic analyses identified a reduction in the branching of hypothalamic astrocytes in the ARC-median eminence junction, suggesting increased blood–brain barrier permeability. These data support the hypothesis that during metabolically challenging conditions like chronic stress, ghrelin may be more able to cross the blood–brain barrier and diffuse throughout the brain to target GHSR-expressing brain regions away from circumventricular organs.

## Significance Statement

Ghrelin is secreted in response to negative energy balance states including stress and is associated with changes in food intake and energy balance. The receptors for ghrelin are found throughout the brain but ghrelin seems to only reach circumventricular regions where the blood–brain barrier is more porous. In this paper, we demonstrate that chronic social defeat stress increases brain permeability to ghrelin to allow for entry and activation of target sites in the mesolimbic dopaminergic system that are not accessible to ghrelin under nonstress conditions. Overall, these results provide an explanation as to how ghrelin can access the mesolimbic dopaminergic system in a state-dependent manner.

## Introduction

Ghrelin is a metabolic hormone primarily produced from X/A-like endocrine cells in the stomach (Kojima et al., 1999). Peripherally produced ghrelin stimulates feeding through activation of the growth hormone secretagogue receptor (GHSR), expressed throughout both the peripheral and central nervous system (CNS; Howard et al., 1996; Zigman et al., 2006). The GHSR is expressed in regions critical for maintaining energy balance and metabolism, including the hypothalamic arcuate nucleus (ARC) and nucleus of the solitary tract (NTS; Zigman et al., 2006), but GHSR expression is also found in midbrain regions, such as the ventral tegmental area (VTA; Abizaid et al., 2006; Zigman et al., 2006), where ghrelin has been shown to regulate dopamine release into the nucleus acumbens and the intake of palatable and rewarding diets (Abizaid et al., 2006; King et al., 2011; Cornejo et al., 2020).

Despite ample expression of the GHSR throughout the CNS, the movement of ghrelin from peripheral circulation into the brain is restricted to circumventricular brain regions where the blood–brain barrier is more porous (Banks et al., 2002; Edwards and Abizaid, 2017). While transport mechanisms have been unveiled for other metabolic hormones, such as insulin, the mode of ghrelin transport is still being elucidated (Edwards and Abizaid, 2017). The median eminence, which sits adjacent to ARC, is a circumventricular organ (CVO), that lacks an intact blood–brain barrier and instead is composed of fenestrated capillaries that allow for the diffusion of larger peptide hormones into the brain (Banks, 2012). The ARC houses neurons sensitive to energy substrates, like glucose or fatty acids, and metabolic hormones, such as ghrelin and leptin. These neurons are at the core of what some have defined as the melanocortin system, a homeostatic system where neurons producing neuropeptide Y and the agouti-related peptide are activated by ghrelin to increase food intake and reduce energy expenditure, and simultaneously inhibit the activity of pro-opiomelanocortin neurons preventing their release of the anorectic peptide α-melanocortin hormone (α-MSH; Cone, 2005). Due to its proximity to the median eminence, ghrelin can easily access the GHSR-expressing cells in the ARC (Schaeffer et al., 2013; Rhea et al., 2018; Fernandez et al., 2023). Beyond this, evidence suggests that ghrelin can be transported throughout the brain via the cerebrospinal fluid (Uriarte et al., 2019); however, the extent to which ghrelin is able to penetrate brain regions, such as the amygdala, hippocampus, and VTA, remains unknown, despite all of these sites expressing the GHSR (Zigman et al., 2006). While peripheral ghrelin fails to reach these sites, intracerebroventricular ghrelin administration induces c-*fos* expression in many of these regions and promotes feeding, confirming that ghrelin acts centrally to convey orexigenic signals (Nakazato et al., 2001; Abizaid et al., 2006). Moreover, ghrelin receptor antagonists delivered into the VTA of rats can prevent peripheral ghrelin-induced food intake (Abizaid et al., 2006).

Chronic psychosocial stressors increase circulating ghrelin and subsequent caloric intake (Lutter et al., 2008; Chuang et al., 2011; Patterson et al., 2013). Mice lacking central GHSR expression do not increase food intake in response to chronic social defeat (Patterson et al., 2013) and show more depressive- and anxiety-like behaviors following this social stress paradigm than WT mice do (Lutter et al., 2008). Thus, it is likely that some of the behavioral outcomes associated with these anxiety and depressive-like behaviors may be related to the protective effects of GHSR signaling in the VTA (Chuang et al., 2011; Park et al., 2021). Nevertheless, it is unclear how elevated circulating ghrelin gains access to the VTA during or following social defeat. One hypothesis is that chronic social defeat can alter permeability in the blood–brain barrier, which would allow increased entry of peripherally secreted hormones, such as ghrelin. Indeed, recent evidence suggest that social defeat results in compromised blood–brain barrier coverage and increased permeability (Menard et al., 2017; Dion-Albert et al., 2022). For example, chronic social defeat stress alters the expression of the tight-junction proteins that comprise the barrier between the brain and vasculature system in association with susceptibility to develop and show depressive-like behaviors (Dudek et al., 2020). Moreover, chronic glucocorticoid treatment results in alterations in the structure and function of astrocytes and tanycytes within regions like the ARC, changes that are linked to obesity (Garcia-Segura et al., 1996; Thaler et al., 2012; Wray et al., 2019). These alterations could also result in increased permeability to peptide hormones, like ghrelin, into regions distant from the CVO-adjacent regions, which may include increased ghrelin entry into stress-responsive regions, like the hippocampus and VTA. Here, we present experiments addressing the hypothesis that chronic social defeat stress produces changes that allow for increased permeability to ghrelin into regions beyond the ARC and median eminence as a potential mechanism that protects against stress-induced pathology.

## Materials and Methods

### Animals

C57BL/6 male mice, aged 8 weeks, were purchased from Charles River Laboratories and used as the experimental mice. These mice were handled daily, and their body weight and food intake were measured every morning at 9:00 A.M. CD-1 male retired breeder mice, aged 24–28 weeks, were also purchased from Charles River Laboratories and used as the aggressive mice for the stress paradigm. All mice were housed in a temperature (24°C)- and humidity (44%)-controlled vivarium. Mice were individually housed at the beginning of the experiment and provided with ad libitum access to laboratory chow and drinking water. All experimental procedures were approved by the Institutional Animal Care Committee (Protocols 119667, 116341, 112032) and followed national guidelines for the ethical use of animals.

### Chronic social defeat stress

Experimental mice underwent a 7 d baseline period during which they were acclimated to being single housed and being handled daily. During this baseline period, food intake and body weight were collected daily. At the end of the baseline period, mice were matched based on body weight and food intake and assigned to one of the following two groups: (1) control no stress (*n* = 24) or (2) stress (*n* = 24). Mice assigned to the stressed group were transferred to a separate room where the CD-1 male resident aggressors were housed and where exposure to social defeat occurred. On the first day of the stressor, the C57BL/6 experimental male mice were each placed into the home cage of a CD-1 resident male mouse. Because male mice, especially larger sexually experienced CD-1 male mice, display aggressive behaviors toward other males intruding in their territory, our CD-1 residents rapidly displayed aggressive behavior toward the experimental C57BL/6 mice placed in their cage. The mice were allowed to interact for no more than 5 min, and as soon as the experimental mice displayed submissive postures as described by Bartolomucci et al. (2004), they were separated to prevent any physical injuries from occurring to the experimental mouse. After the interaction period, the mice were separated by a transparent, mesh divider and housed in the same cage for the next 24 h. The divider prevented any physical contact or harm but allowed continual sensory contact between the mice. The following day, the experimental mice were removed from teach cage and introduced into the cage of a new CD-1 mouse. The interaction was repeated daily, at 10:00 A.M., for 21 d.

### Ghrelin administration

Twenty-four hours after the final stress exposure, the mice were injected subcutaneously with 300 pmol/g BW of [Dpr3(octanoyl),Lys19(sulfoCy5]-ghrelin(1–19) (Cy5-ghrelin), a peptide analog of acyl-ghrelin peptide with a sulfo-Cyanine5 molecule attached to the Lysine19 at the C terminus. This analog has been previously confirmed to behave like endogenous acyl-ghrelin in GHSR binding (McGirr et al., 2011; Douglas et al., 2014; Childs and Luyt, 2020). Cy5-ghrelin was synthesized and purchased from Dr. Luyt at The University of Western Ontario. The peptide was of >99% purity in the form of trifluoroacetate salt. Cy5-ghrelin was dissolved in sterile saline on the morning of injection. The dose used was chosen based on (1) a previously reported dose to visualize fluorescence within the mouse central nervous system (Uriarte et al., 2019) and (2) a dose that closely resembles circulating ghrelin in mice exposed to chronic social defeat stress (Chuang et al., 2011). Mice were killed 7, 15, 30, or 60 min after the injection by intracardial perfusion with ice-cold 0.1 M of phosphate-buffered saline (PBS) followed by 4% paraformaldehyde under isoflurane anesthesia. Vehicle-treated mice were killed 30 or 60 min postinjection and were used as a negative control group to which Cy5-ghrelin quantifications were normalized.

### Tissue processing

Following perfusion, brains were extracted and postfixed in paraformaldehyde for 24 h, before being transferred to a 30% sucrose solution for at least 48 h for cryoprotection. Once ready, brains were frozen and sliced on a cryostat. The sections were collected into four series of 40 µm sections that were stored in a Watson's cryoprotectant solution (Watson et al., 1986) at −20°C until they were processed for immunocytochemistry and microscopy.

### Immunohistochemistry and quantification of ghrelin fluorescence

Brain sections from Cy5-ghrelin or vehicle-injected mice were used to visualize and measure the movement of ghrelin into and throughout the brain. To enhance the signal, sections were processed for immunohistochemistry using an anti-Cy5 antibody. In short, brain sections were washed with 0.01 M PBS and incubated for 1 h in blocking buffer solution (1% BSA, 3% normal goat serum, 0.5% Triton X-100, 0.01 M PBS), followed by 48 h in mouse anti-Cy5 antibody (Millipore, catalog #1117, 1:2,500) at 4°C. Sections were then washed in PBS and incubated with goat anti-mouse Alexa Fluor 594 nm (Invitrogen, catalog #A-11005, 1:250) for 2 h at room temperature. Negative controls with the absence of either the primary or secondary antibodies were included to validate the staining protocol. Sections were mounted and coverslipped using antifaded fluorescent mounting media (Millipore, catalog #HC08) and stored at room temperature, protected from light. Fluorescent microscope images were obtained on a semiconfocal spinning disk microscope (Olympus BX61) using Invitro3 imaging software. Fluorescence quantification analyses were performed using Fiji on images obtained at 20× magnification, using identical settings to capture all images. Images were quantified by an experimenter blind to the treatment conditions of each animal. The regions of interest were identified using the Allen Reference Atlas (2008) and sections from the corresponding bregma level for each region {NAc, 1.245–0.62; hypothalamus [ARC, VMH, dorsomedial hypothalamus (DMH), LH], −1.355 to −1.855; dentate gyrus, −1.655 to −2.055; VTA, −2.88 to 3.38}. We also examined differences in fluorescence in regions like the organum vascularis of the stria terminalis (OVLT; 0.4–0.7), subfornical organ (SFO; −0.1 to −0.5), and choroid plexus (CP; −0.3 to −0.7), but these were not quantified due to lack of sufficient sections to have enough power to do so. Background fluorescence due to spatial disparities and endogenous fluorescence were removed using Fiji. The fluorescent intensity of the Cy5-ghrelin signal was quantified by measuring integrated density, the sum of pixel intensity relative to the area measured. Measurements are expressed as the change in fluorescent intensity, relative to the signal obtained from vehicle-treated control animals.

### c-*fos* immunohistochemistry

Mice that were killed 60 min following injection (vehicle, *n* = 6; Cy5-ghrelin, *n* = 12) were used to investigate Cy5-ghrelin–induced neuronal activation using c-*fos* immunocytochemistry. Brain sections were washed with 0.01 M PBS before incubation in 1% hydrogen peroxide (in PBS) for 30 min. Sections were then incubated in blocking buffer (1% BSA, 3% normal goat serum, 0.5% Triton X-100, 0.01 M PBS) followed by 48 h in rabbit Fos antibody (aFos ab-5, 1:20,000, Oncogene Research Products) in a blocking buffer at 4°C. Sections were then washed with PBS and incubated with biotinylated donkey anti-rabbit antibody (Jackson ImmunoResearch, catalog #711-067-003, 1:250 in PBS) for 2 h at room temperature. To enhance the signal, sections were incubated in ABC solution (VectorLabs, catalog #PK-6100) for 1 h followed by a DAB solution (DAB, Sigma-Aldrich, catalog #91-95-2, 0.05%) of cobalt chloride (0.05%) and nickel ammonium sulfate (0.05%) to produce a black precipitate in c-*fos*^+^ cells. Sections were then mounted onto glass slides and dehydrated using a series of EtOH and xylene (Millipore, catalog #108298), before being coverslipped (Thermo Fisher Scientific, catalog #4111). Microscope images were obtained using a Zeiss AxioPlan light microscope on 10× magnification. Images were analyzed by an experimenter who was blind to the treatment conditions of each animal. The regions of interest were identified using the Allen Reference Atlas (Dong, 2008) and sections from the corresponding bregma level for each region [NAc, 1.245–0.62; hypothalamus (ARC, VMH, DMH, LH), −1.355 to −1.855; dentate gyrus, −1.655 to −2.055; VTA, −2.88 to 3.38]. The number of activated cells was quantified using Fiji. Images were converted to 8-bit grayscale images and the threshold was adjusted to remove the background and only display c-*fos*+ cells. First, the region of interest was outlined using the “polygon” function, and the area of interest in microns was measured. Then the “watershed” function was used to delineate the borders of cells, and the “analyze particles” function was used to automatically identify and count cells within the designated area. The area measured per each region of interest did not differ significantly between the groups analyzed. The results displayed express the cumulative number of c-*fos*+ cells counted in each region of interest.

### Astrocyte immunohistochemistry and quantification

A subset of brain sections (*n* = 5/group, nonstress or stress) were selected to be used for the investigation of hypothalamic astrocytes. Immunohistochemistry for glial fibrillary acidic protein (GFAP) was performed following the same protocol as described above with the following antibody modifications. Sections were incubated in rabbit anti-GFAP antibody (Abcam, catalog #ab7260, 1:1,000) and donkey anti-rabbit Alexa Fluor 488 nm (Invitrogen, catalog #A21206, 1:250), followed by Hoechst 33258 (Sigma-Aldrich, #94403, 1:15,000) for 2 min. Images were collected at 40× magnification in a *z*-stack, and the Sholl analysis was performed using the neuroanatomy Fiji plug-in. The images were converted to 8-bit and optimized for brightness contrast before thresholding to minimize background noise. The ARC from bregma −1.45 to −1.85 mm was used in the analysis to ensure consistency of ROI, with *n* = 6 astrocytes measured per mouse. To ensure the accuracy of measurements, only nonoverlapping GFAP+ cells were analyzed. The Sholl analysis was performed using SNT (Arshadi et al., 2021), and concentric circles were placed on the 2D astrocyte images, starting at the thickest point in the soma and radiating outward to the tip of the longest projection. Each circle was placed an equal distance apart from one another (step size), 2 µm, that was chosen based on the size of the astrocytes in this region. The number of intersections per step size was defined as the number of times processes cross each delineated circle.

### Dextran immunohistochemistry

An additional cohort of mice (*n* = 4/group, nonstress or stress) was injected with Alexa Fluor 488 dextran to evaluate blood–brain barrier leakiness, as described by Dion-Albert et al. (2022). Here, 24 h after the final stress exposure mice were anesthetized and administered 0.1 ml of 1 mg/ml of Alexa Fluor 488 dextran (D22910, 10 kDa, Thermo Fisher Scientific) via retro-orbital injection. Mice were perfused 30 min later, and tissues were processed as described above. Following slicing, sections were incubated in a blocking buffer for 1 h followed by *Lycopersicon esculentum* (tomato) lectin (1:400, L32471, Thermo Fisher Scientific) for 5 min, protected from light. The tissues were then washed with PBS before mounting and coverslipping. Slides were then imaged semiconfocal spinning disk microscope (Olympus BX61) using cellSens Dimension. Background fluorescence due to spatial disparities and endogenous fluorescence were removed using Fiji to detect the mean fluorescent intensity of Alexa Fluor 488 dextran in the median eminence and ARC sections.

### Statistical analyses

Food intake and body weight data of the control and stressed mice were analyzed using unpaired *t* tests. Fluorescent intensity of Cy5-ghrelin in nonstressed and stressed mice was analyzed using a two-way ANOVA, to determine differences between subjects (nonstress vs stress) over time. The normality of variance assumption was assessed using the Shapiro–Wilk test, with a *p* > 0.05 considered to be normally distributed, and justified the use of parametric analyses. Tukey's test for post hoc comparisons was used to determine if there were significant differences between the groups in the case of significant interaction effects. The c-*fos* data were analyzed using a one-way ANOVA to determine differences relative to vehicle-injected controls. The vehicle-injected control groups were pooled (nonstress and stress) due to nonsignificant differences between groups. For the Sholl analysis, a mixed-effect repeated measure ANOVA was used to analyze the number of intersections per radius step. All data is expressed as mean ± SEM. GraphPad Prism was used for analyses.

## Results

### Chronic social defeat stress increases ghrelin entry to hypothalamic and extrahypothalamic regions

**Figure 1.**
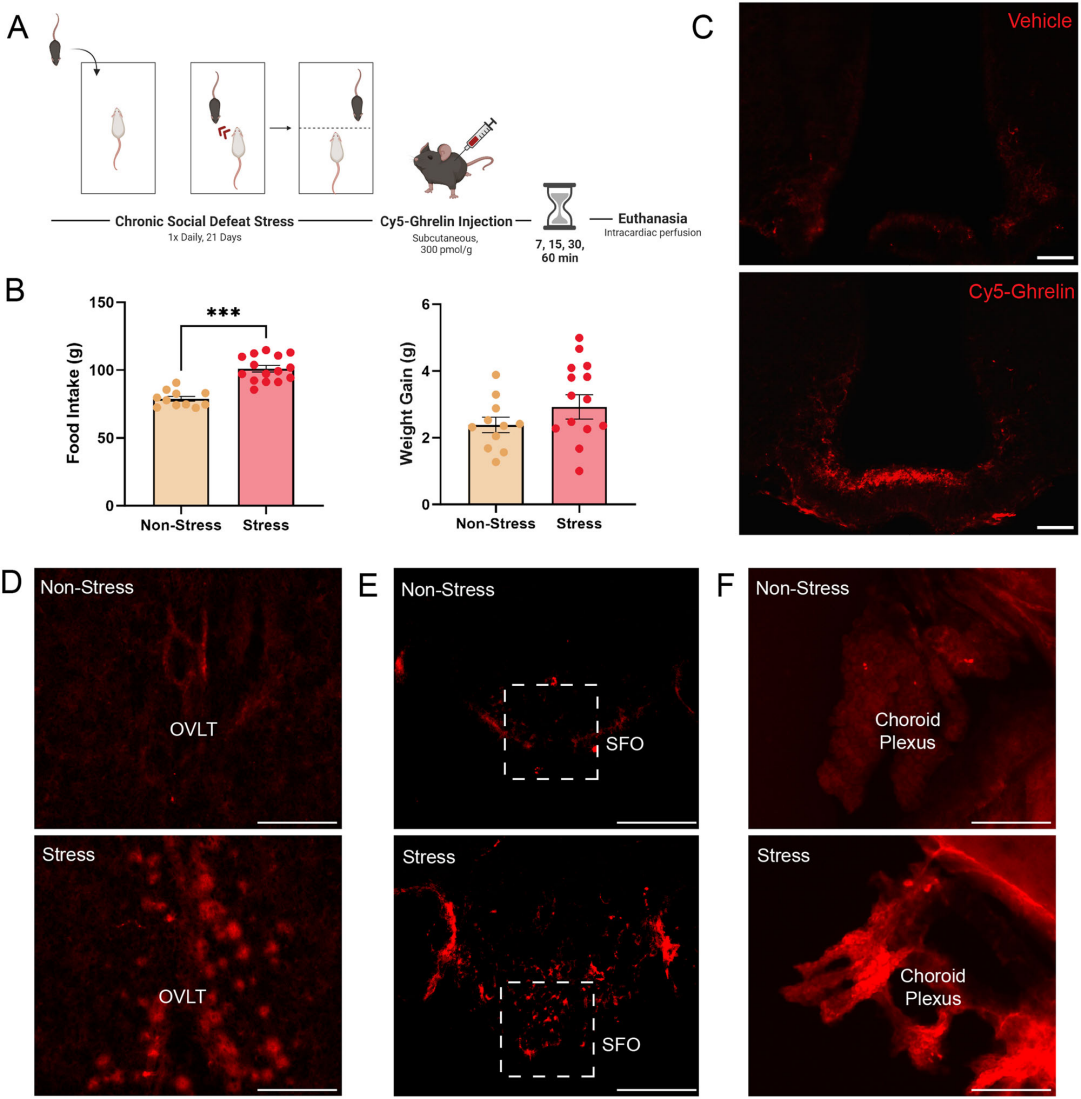
Peripherally administered Cy5-ghrelin accesses the brain via median eminence. ***A***, Experimental timeline of 21 d chronic social defeat stress paradigm, followed by timed Cy5-ghrelin administration and perfusion. ***B***, Chronic social defeat stress increased total food consumed during the stress paradigm but did not alter weight gain. ***C***, Validation of Cy5-ghrelin labeling revealed similar patterns of entry via the median eminence, which was unique from vehicle-injected controls. Fluorescent intensity from vehicle-injected controls was used to normalize the Cy5-ghrelin fluorescence results. Panels ***D–F*** show Cy5-ghrelin signal in the OVLT (panels ***a*** and ***b***), SFO (panels ***c*** and ***d***), and choroid plexus (CP; panels ***e*** and ***f***) of control and socially defeated mice. As shown in this image, mice exposed to chronic social defeat appeared to have increased Cy5-ghrelin signal compared with signal in the same regions compared with nonstressed mice. Scale bars: ***C***, 100 μm; ***D***–***F***, 50 μm. ***p < 0.001.

Figure 1 shows food intake and weight gain in control mice and mice exposed to 21 d of chronic social defeat stress (Fig. 1*A,B*). As shown in this figure, socially defeated mice increased their food intake (*t*_(24)_ = 6.867, *p* < 0.001), compared with the stress-naive counterparts, although this increase in food intake did not result in an increase in significant weight gained by the end of the experiment (*t*_(24)_ = 1.147, *p* = 0.263; Fig. 1*B*). Vehicle-injected mice showed very low levels of background fluorescence compared with Cy5-ghrelin injected mice in which fluorescence signal was detected in the median eminence and mediobasal hypothalamus including the ARC (Fig. 1*C*). As shown in Figure 1, *D–F*, stressed mice showed increased ghrelin-Cy5 signal in circumventricular organs like the OVLT, SFO, and CP although we did not have sufficient sections to analyze fluorescence intensity differences between groups.

**Figure 2.**
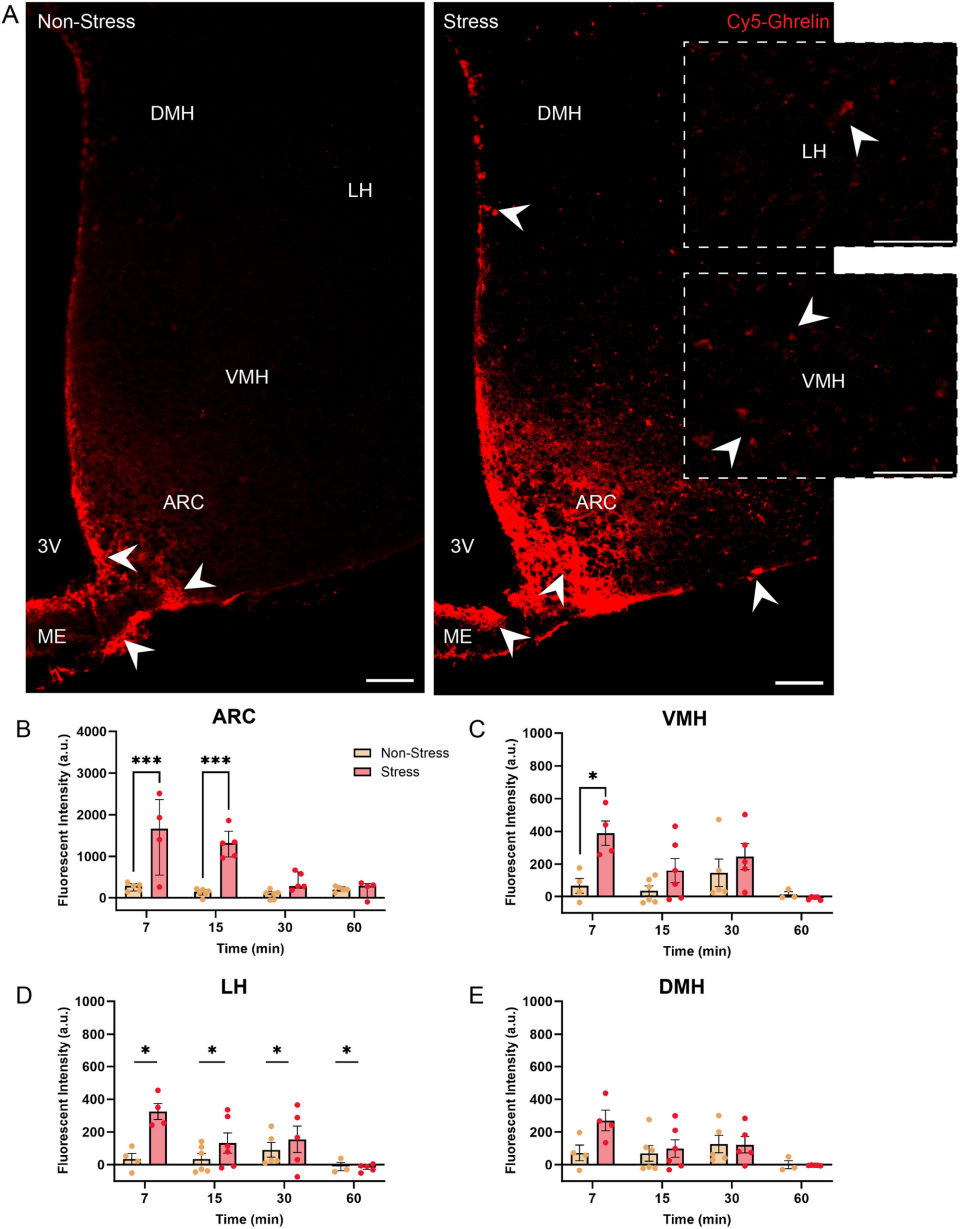
Chronic social defeat stress increases detectable ghrelin signals in hypothalamic nuclei. ***A***, Cy5-ghrelin fluorescent signal 7 min following peripheral injection shows increased ghrelin infiltration into the hypothalamus in stressed mice. The arrowheads highlight the areas of Cy5-ghrelin labeling in nonstressed and stressed mice. Scale bar, 100 µm. Cy5-ghrelin fluorescent signal was elevated in stressed mice in the (***B***) ARC at 7 and 15 min following injection and in the (***C***) VMH at 7 min following injection. ***D***, Cy5-ghrelin fluorescent intensity was increased in the LH of stressed mice, regardless of time since injection. ***E***, There were no significant differences in the DMH. *p < 0.05, **p < 0.01, ***p < 0.001.

As shown in Figure 2, we examined and quantified differences in Cy5-ghrelin fluorescent signal throughout regions of the mediobasal hypothalamus including the ARC, VMH, DMH, and LH (Fig. 2). As shown in this figure, Cy5-ghrelin fluorescent intensity in the ARC (*F*_(3, 32)_ = 8.04, *p* < 0.001, *η*^2^ = 0.19) and VMH (*F*_(3, 30)_ = 3.150, *p* = 0.048, *η*^2^ = 0.12) increased in chronically stressed mice over the time course evaluated, compared with nonstressed controls. In the ARC, stressed mice exhibited significantly higher Cy5-ghrelin signal at 7 and 15 min following injection (*p* < 0.001), compared with nonstressed counterparts at the same time points (Fig. 2*B*). Meanwhile in the VMH, Cy5-ghrelin fluorescence was elevated in stressed mice 7 min following injection (*p* = 0.039), compared with nonstressed counterparts (Fig. 2*C*). In the LH, Cy5-ghrelin fluorescence was increased in the stressed mice, regardless of time (main effect of stress; *F*_(1, 30)_ = 9.192, *p* = 0.005, *η*^2^ = 0.15; Fig. 2*D*). Finally, we found that Cy5-ghrelin signal in the DMH differed across time (main effect of time; *F*_(1.9, 12.8)_ = 4.35, *p* = 0.037, *η*^2^ = 0.23), but stressed mice did not show increases in the amount of fluorescent ghrelin signal observed compared with nonstressed mice (*p* > 0.05; Fig. 2*E*).

**Figure 3.**
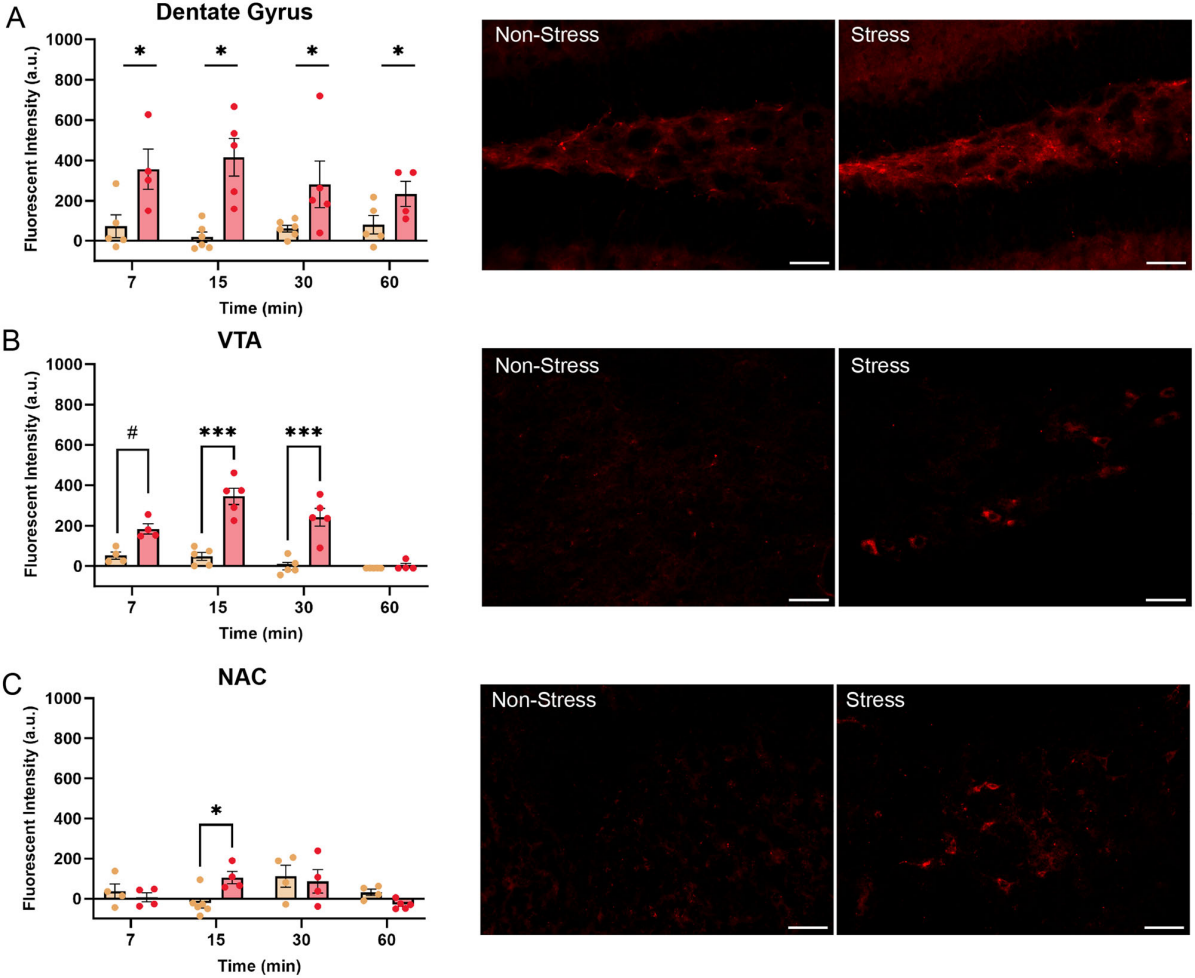
Chronic social defeat enhances detectable ghrelin signals in nonhypothalamic, ghrelin-responsive brain regions. ***A***, Stress increased Cy5-ghrelin fluorescent signal in the dentate gyrus of the hippocampus, which was not altered by time following injection. ***B***, Cy5-ghrelin signal was nearing a significant increase into the VTA at 7 min and was significantly elevated at 15 and 30 min, as displayed in microscopy images. ***C***, Cy5-ghrelin fluorescent intensity in the NAc increased in stressed mice at 15 min following injection. Images show Cy5-ghrelin signal 15 min following peripheral administration. Scale bar, 50 µm. ^#^*p* < 0.07, *p < 0.05, ***p < 0.001.

We then quantified and examined differences in Cy5-ghrelin fluorescent signal in nonhypothalamic brain regions that express the GHSR. As shown in Figure 3, we found that Cy5-ghrelin fluorescence in the dentate gyrus of stressed mice was significantly higher than nonstressed mice (main effect; *F*_(2, 32)_ = 29.71, *p* < 0.001, *η*^2^ = 0.44), which was still evident 60 min following injection (Fig. 3*A*). The GHSR has been detected in the VTA and the nucleus accumbens, both hubs of the mesolimbic reward system (Abizaid et al., 2006; Zigman et al., 2006). Examination of Cy5-ghrelin signal in the VTA revealed that Cy5-ghrelin fluorescence was significantly increased in socially defeated mice, compared with controls (*F*_(3, 29)_ = 11.07, *p* < 0.001, *η*^2^ = 0.16; Fig. 3*B*). Here, fluorescent intensity was increased at 15 (*p* < 0.001) and 30 min (*p* < 0.001) after the ghrelin was injected. Meanwhile, in the NAc, Cy5-ghrelin signal was significantly altered (*F*_(3, 27)_ = 3.3, *p* = 0.035, *η*^2^ = 0.21) between nonstressed and stressed mice at 15 min following injection (*p* = 0.039; Fig. 3*C*).

### Stress increases ghrelin-induced neuronal activation in the mediobasal hypothalamus and VTA

**Figure 4.**
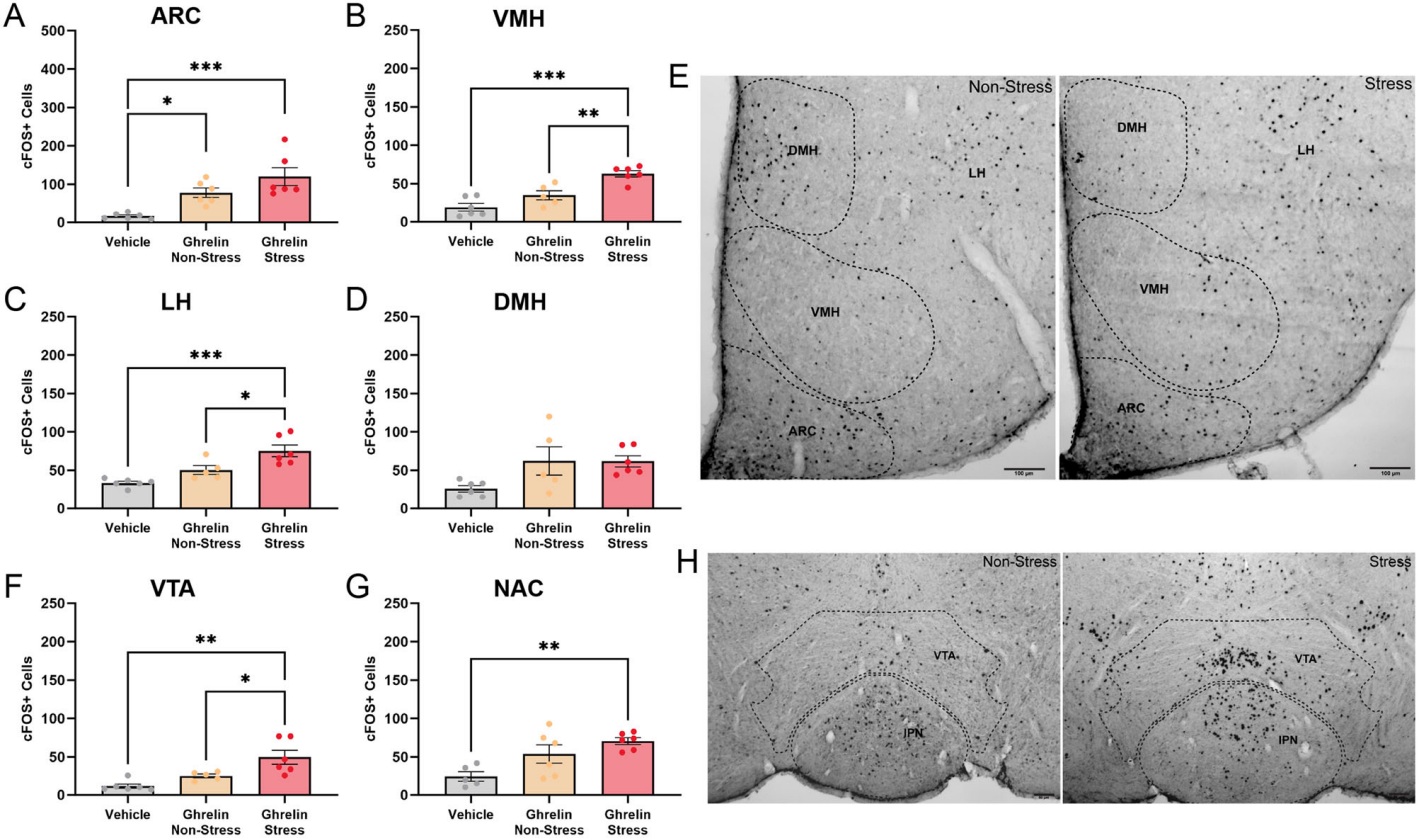
Ghrelin increases c-*fos* expression throughout the mediobasal hypothalamus and midbrain of socially defeated mice. ***A***, Ghrelin increased the number of c-*fos*+ cells in the ARC of both nonstressed and stressed mice. Socially defeated mice had an increase in c-*fos*+ cells in the (***B***) VMH and (***C***) LH in response to ghrelin administration. ***D***, There was no difference in c-*fos*–expressing cells in the DMH. ***E***, Images of the mediobasal hypothalamus highlight stress-related changes in ghrelin-induced c-*fos* expression. Scale bar, 100 µm. ***F***, Stress enhanced ghrelin's activation of c-*fos* in the VTA, while in the (***G***) NAc, ghrelin increased c-*fos*+ cells compared with vehicle-injected controls. ***H***, Midbrain images highlight differences in c-*fos* expression in the VTA of nonstressed and stressed mice. Scale bar, 50 µm. *p < 0.05, **p < 0.01, ***p < 0.001.

The increase in ghrelin signal observed in socially defeated mice would suggest that ghrelin can enter the brain and have functional effects in the regions investigated. To determine if this is the case, we examined neuronal activation as determined by increases in the number of c-*fos*–expressing cells following injections of saline or Cy5-ghrelin in stressed and nonstressed mice. As shown in Figure 4, Cy5-ghrelin injections increased the number of c-*fos*+ cells in the ARC (*F*_(2, 15)_ = 11.93, *p* < 0.01, *η*^2^ = 0.61) in nonstressed (*p* = 0.028) and stressed (*p* < 0.001) mice, compared with the vehicle-injected controls (Fig. 4*A*). There was, however, no difference in number of c-*fos*–expressing cells between the nonstressed and stressed mice. In the VMH, the stressed Cy5-ghrelin–treated mice showed an increase in c-*fos*–expressing cells (*F*_(2, 14)_ = 20.44, *p* < 0.001, *η*^2^ = 0.74), compared with vehicle-injected mice (*p* < 0.001). This increase in c-*fos* was significantly higher than that observed in nonstressed Cy5-ghrelin–injected mice (*p* = 0.005; Fig. 4*B*). As shown in Figure 4*E* images, this appeared to be due to an increase in c-*fos*+ cells in the central and ventrolateral subregions of the VMH. Cy5-ghrelin also induced an increase in c-*fos* expression in the LH of the stressed mice (*F*_(2, 14)_ = 14.67, *p* < 0.001, *η*^2^ = 0.67), in comparison with vehicle-injected controls (*p* < 0.001; Fig. 4*C*). There was also a significant increase in c-*fos*–expressing cells in stressed mice, compared with nonstress counterparts (*p* = 0.02). Meanwhile, there were no significant differences in c-*fos* expression in the DMH between the groups (*p* > 0.05).

When evaluating the other regions that displayed changes to Cy5-ghrelin accumulation, Cy5-ghrelin increased the number of c-*fos*–expressing cells in the VTA (*F*_(2, 14)_ = 10.93, *p* = 0.001, *η*^2^ = 0.60) in the stressed mice, compared with the vehicle-injected controls (*p* = 0.001) and nonstressed counterparts (*p* = 0.035; Fig. 4*F,H*). In the NAc, ghrelin also induced an increase in c-*fos*+ cells (*F*_(2, 14)_ = 7.34, *p* = 0.007, *η*^2^ = 0.51; Fig. 4*G*), where stressed mice exhibited an increase in c-*fos*–expressing cells, compared with vehicle-injected controls (*p* = 0.005); however, there were no differences compared with the nonstressed mice. Cy5-ghrelin had no effect on c-*fos* expression in the dentate gyrus (data not shown).

### Stress induces morphological changes in the mediobasal hypothalamus

**Figure 5.**
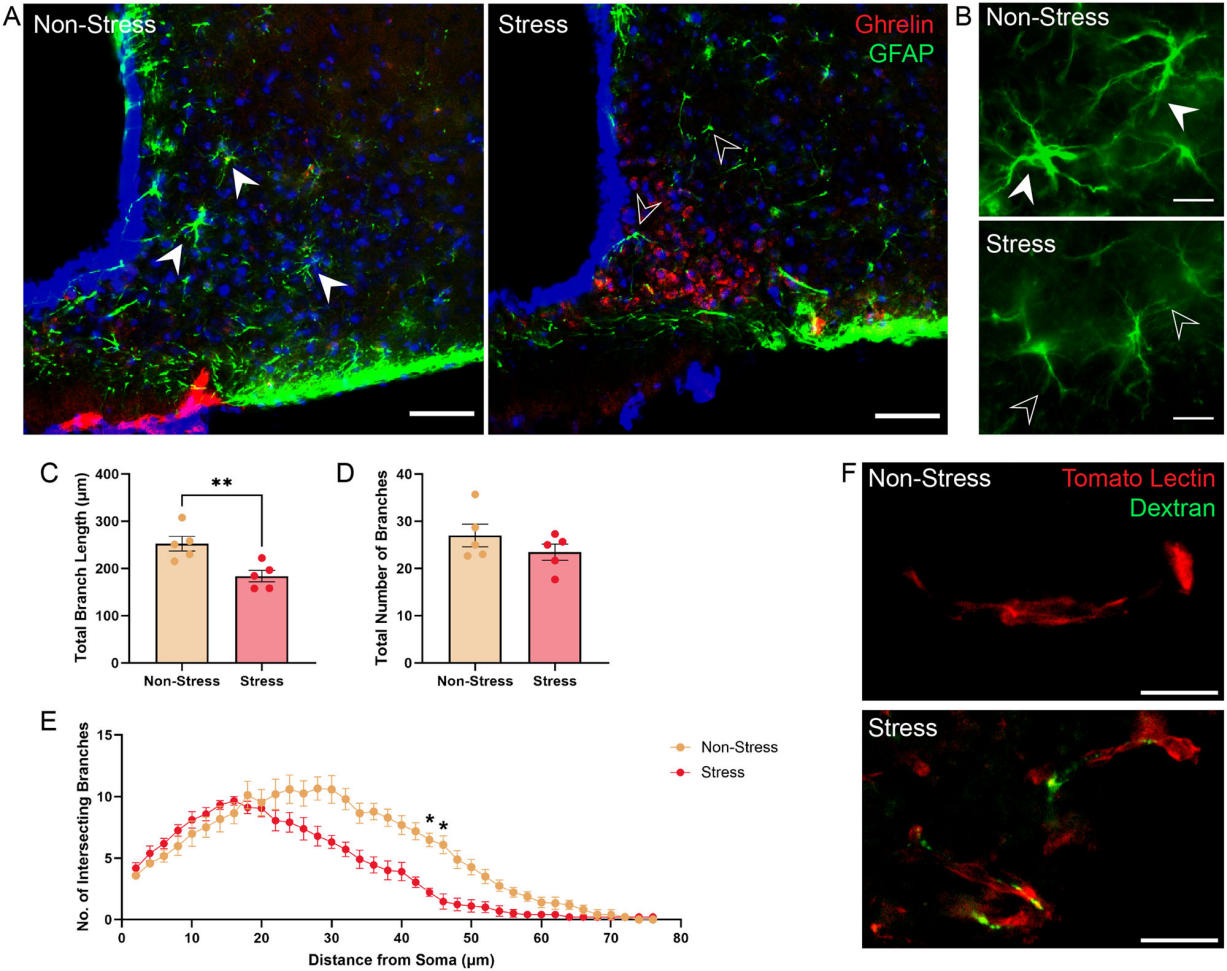
Chronic stress induces morphological changes to astrocytes in the ARC surrounding areas of ghrelin entry. ***A***, ***B***, Images highlight the changes in astrocyte morphology including a reduction in the number of branches and branch length (empty arrowhead) in stressed mice, compared with nonstressed controls that displayed extensive branching and radial morphology (filled arrowhead). Reduction in astrocyte branching was identified near the Cy5-ghrelin signal. Scale bar, 100 µm. ***C***, Cumulative branch length of astrocytes in stressed mice was significantly decreased, compared with nonstressed controls. ***D***, There was no difference in the total number of astrocyte branches between the stressed and nonstressed groups. ***E***, Sholl analysis revealed a significant difference in the number of astrocyte branch intersections measured at a constant radial distance from the cell body, with stressed mice exhibiting a significant reduction in branches at 44 and 46 µm from the soma. ***F***, Dextran fluorescent signal in neurovasculature (tomato lectin) of the ARC was visible in stressed mice. ***G***, Chronic stress was associated with an increase in the mean fluorescent intensity in the median eminence and ARC junction, compared with nonstressed controls, suggesting leakiness of the blood–brain barrier. *p < 0.05, **p < 0.01.

One way in which stress may facilitate ghrelin entry into the brain is via alterations in the blood–brain barrier as previously reported (Menard et al., 2017). To examine if this occurred in the hypothalamus, we evaluated the morphology of astrocytes at the intersection of the median eminence and the ARC, given that these have been implicated in the entry of metabolic hormones such as leptin (Pan et al., 2008) and due to their close connection with the endothelial cells that compromise the blood–brain barrier and surrounding vascular space (Cheunsuang and Morris, 2005; Chowen et al., 2016). As highlighted in Figure 5, immunohistochemistry of GFAP cells in the junction between the median eminence and ARC revealed differences in cell morphology, between the nonstressed and stressed mice. In socially defeated mice, the astrocytes lacked the star-like, radial projections that are normally associated with these cells and were, instead, flatter and with fewer projections (Fig. 5*A,B*). These morphological changes were quantified using a Sholl analysis that measures the length and number of branches stemming from the GFAP+ cell bodies. The Sholl analyses revealed a reduction in the total length of astrocytic branches measured in the chronically defeated mice, compared with those of nonstressed controls (*t*_(8)_ = 3.436, *p* = 0.009, *η*^2^ = 0.59; Fig. 5*C*). Astrocytic branches were measured at a radial distance every 2 µm from the cell body and these analyses revealed that the number of branches differed significantly (*F*_(37, 296)_ = 6.35, *p* < 0.001, *η*^2^ = 0.44) between the nonstressed and stressed mice (Fig. 5*E*). Results from these analyses indicate that astrocytes in the ARC of stressed mice had fewer branches as distance from the cell body increased, and the number of branches was significantly lower in the stressed mice at a radius of 44 µm (*p* = 0.015) and 46 µm (*p* = 0.048) away from the cell body, compared with the number of branches in the nonstressed mice at these distances. To determine if the changes in astrocyte morphology impacted blood–brain barrier coverage, socially defeated mice were injected 24 h after their last stress session with a fluorescently tagged dextran that is unable to cross the blood–brain barrier under normal conditions and examined if this signal was present in the ME/ARC of these mice in comparison with similarly injected nonstressed mice. As shown in Figure 5*F*, we found that there was no observable dextran signal in the nonstressed control mice; however, we identified dextran accumulation in blood vessels in the ARC of stressed mice. There was a significant increase in fluorescent dextran signal in the ARC blood vessels of stressed mice (*t*_(6)_ = 4.716, *p* = 0.003, *η*^2^ = 0.79), compared with nonstressed controls (Fig. 5*G*), suggesting the presence of leaky blood vessels in this area.

## Discussion

Under normal conditions, peripheral ghrelin has limited access to the brain, with CVOs like the median eminence being a primary route of entry (Cabral et al., 2013; Uriarte et al., 2019). Despite this, ghrelin receptors are found in regions of the brain in which circulating ghrelin has restricted access, including the VTA and NAc, areas where ghrelin can bind to regulate reward-seeking behaviors (Naleid et al., 2005; Abizaid et al., 2006; Skibicka et al., 2011; King et al., 2011). Our results provide evidence that chronic stress results in increased permeability in the blood–brain barrier in the median eminence and ARC, and this facilitates the entry of ghrelin into the hypothalamus and the distribution of this hormone to other regions in the brain, including areas associated with the regulation of motivation and emotion.

Chronic social defeat stress increases caloric intake and the secretion of ghrelin in male and female mice (Chuang et al., 2011; Patterson et al., 2013; Smith et al., 2023). Importantly, these increases have been linked to GHSR expression in the VTA, a region that is important for the regulation of motivated states, like reward-seeking and stress response (Lutter et al., 2008; Chuang et al., 2011; King et al., 2011). In this paper, we show that, as in previous works, chronic social defeat stress increased food intake. Importantly, we found that this effect was associated with an increase in Cy5-labeled ghrelin in the median eminence and ARC. The elevated Cy5-ghrelin fluorescence in the ARC and median eminence indicates that, despite the fact that the secretion of endogenous ghrelin increases after chronic social defeat stress in mice (Lutter et al., 2008; Patterson et al., 2013; Smith et al., 2023), Cy5-ghrelin is entering this region with more ease after 3 weeks of social defeat stress than it is in nonstressed mice. Overall, this would suggest that brain permeability to ghrelin in the mediobasal hypothalamus is increased in chronically stressed mice.

Despite the increase in Cy5-ghrelin signal in the ARC, the number of c-*fos*–expressing cells in the ARC after the Cy5-ghrelin injection was equally increased in stressed and nonstressed mice. This may be due to the fact that while ghrelin secretion and entry in the brain may be increased, GHSR expression in the ARC of socially defeated male mice does not change in response to social defeat stress (Patterson et al., 2013; Smith et al., 2023). One possibility that could explain this result is that the binding sites for ghrelin are fully occupied in the ARC, resulting in unbound ghrelin being transported to other hypothalamic and nonhypothalamic regions. Indeed there is evidence showing that ghrelin is transported to other regions of the brain via the blood–CSF system (Cabral et al., 2013; Uriarte et al., 2019) allowing ghrelin to impact metabolic rates and glucose metabolism in a wide range of brain regions (De Francesco et al., 2023). This is supported by the difference in the time in which the Cy5-labeled ghrelin signal peaked in the hypothalamus (7 min postinjection) versus regions like the VTA, NAc, and hippocampus (15 min postinjection). Importantly, the increase in signal observed in the ARC was also detected in other circumventricular regions including the choroid plexus, a region where cerebrospinal fluid is produced. Overall, this supports the notion of increased brain permeability to ghrelin and potentially other blood-borne hormones following chronic social defeat.

In contrast, chronic social defeat stress did not seem to increase fluorescent ghrelin binding nor c-*fos* expression in the DMH of chronically stressed mice, potentially indicating that stress does not impact the ability to bind or stimulate the DMH, a region that can be activated by ghrelin to regulate feeding and energy expenditure (Hyland et al., 2020). Within the VMH, the increase in fluorescent ghrelin binding and c-*fos* expression were primarily observed in the ventrolateral portion, a subregion in the VMH that is linked to social behaviors that include mating and aggression in mice, and not with metabolic function (Lin et al., 2011; Falkner et al., 2016; Flanigan and Russo, 2019). Ghrelin has been linked to behavioral responses with a social component, and some have suggested that ghrelin may play a role in maintaining social behaviors (Park et al., 2021; Pfabigan et al., 2024), and resilience in general, in the face of significant stressors like the stress experienced by socially defeated mice (Lutter et al., 2008).

Another region that may be important in maintaining resilience following a chronic social defeat is the VTA (Friedman et al., 2014; Koo et al., 2019), a region where GHSR is expressed (Zigman et al., 2006) and where Cy5-ghrelin signal was increased in socially defeated mice. The VTA displays relatively high levels of GHSR expression in rats and mice, and ghrelin has the capacity to stimulate dopamine cells in this region and the release of dopamine in the NAc, in association with increases in feeding and food motivation (Abizaid et al., 2006; King et al., 2011). Nevertheless, peripheral ghrelin has difficulty reaching the VTA or the NAc, at least under regular conditions or following an acute fast (Cabral et al., 2014). As shown by our data, Cy5-ghrelin labeling in the VTA was significantly higher in stressed mice 15 min after injection and returned to baseline levels 60 min following injection. The VTA also displayed increased c-*fos* expression in response to Cy5-ghrelin in stressed mice, compared with nonstressed and vehicle-injected controls. This supports previous research highlighting that chronic social defeat stress increased GHSR expression in the VTA of defeated male mice, suggesting that ghrelin activity in this region is enhanced following stress exposure (Chuang et al., 2011; Smith et al., 2024). Additionally, Cy5-ghrelin labeling was also increased in the NAc at the 15 min time point, and c-*fos* expression was increased 60 min following Cy5-ghrelin injection, compared with vehicle-injected controls. The increase in c-*fos* expression may be attributed to increased activity of VTA afferents to the nucleus accumbens and potentially direct effects of ghrelin on VTA presynaptic terminals in the nucleus accumbens (Abizaid, 2009). These data also suggest that GHSR-expressing neurons in the VTA and terminals in the NAc respond to peripheral ghrelin under chronically stressful conditions. Interestingly, our results also show that Cy5-ghrelin labeling in the dentate gyrus of the hippocampus was elevated in stressed mice, regardless of the time points examined. Nevertheless, and despite this elevation in Cy5-ghrelin labeling, stressed mice did not show increased c-*fos* expression in the dentate gyrus compared with nonstressed mice, suggesting that additional ghrelin entry into this region may have effects that are independent of the activation of c-*fos* and perhaps may target these cells and stimulate them via alternate cellular signaling pathways (Kanoski et al., 2013).

Ultimately, these data suggest that chronic social defeat alters the integrity of the blood–brain barrier (Dion-Albert et al., 2022) and that ghrelin capitalizes on this effect. Indeed, previous work has demonstrated that chronic social defeat stress disrupts the integrity of the blood–brain barrier in regions like the NAc (Menard et al., 2017). The blood–brain barrier is comprised of tight-junction enclosed endothelial cells, which are surrounded by astrocytic end feet. The astrocytes aid in bidirectional communication with surrounding neurons, can regulate blood–brain barrier permeability, and contribute to blood–brain barrier pathology (Abbott et al., 2006). In the ARC we found that GFAP+ astrocytes of chronically stressed mice displayed morphological changes, including a reduction in the radial projections stemming from the soma and length of said projections. This suggests a retraction in the astrocytic end feet that normally encapsulates the endothelial cells, an effect that may contribute to leakiness of the neurovasculature in this region, leading to the increased entry of Cy5-ghrelin that was observed. Supporting this effect, we found that peripheral injection of fluorescently labeled dextran that cannot readily cross an intact blood–brain barrier resulted in increased fluorescent dextran infiltration surrounding the vasculature of the ARC in socially defeated mice. Therefore, the reduction in length and branching of astrocytic end feet may have increased leakiness in the blood–brain barrier, due to incomplete coverage of the endothelial cells.

These changes may not be unique to chronic social defeat. Indeed, astrocytes are responsive to other challenges to energy balance and reproduction (Pinto et al., 2004; Garcia-Segura et al., 2008; Chowen et al., 2016), and exhibit changes in morphology in response to the feeding status of laboratory rodents (Pinto et al., 2004; Lee et al., 2012). For example, fasting or overconsumption of a high-fat diet induces changes in astrocyte morphology and GFAP expression in the hypothalamus (Thaler et al., 2012; Y. Zhang et al., 2017; Varela et al., 2020). It is also possible that the changes observed are caused by glucocorticoids, given that these are commonly elevated during fasting (Makimura et al., 2003), high-fat diet (Thaler et al., 2012), or chronic social defeat stress exposure (Patterson et al., 2013). Astrocytes have been shown to be sensitive to the effects of glucocorticoids (Bohn et al., 1991), and glucocorticoids can inhibit astrocyte growth (Crossin et al., 1997). Stressor length and, thus, duration of glucocorticoid exposure may play a role in long-term changes to neurovascular cells, leading to the changes we observed here. Additionally, previous work demonstrated that social stress–induced changes to the expression of tight-junction proteins in the endothelial cells lead to leakiness of the blood–brain barrier (Menard et al., 2017). While we did not evaluate the tight junctions in this study, our results support that changes in astrocyte morphology are sufficient to induce changes in blood–brain barrier permeability.

Several other cell types comprising the blood–brain barrier may have been impacted by the stressor and therefore, contributing to ghrelin movement into the brain. For instance, vascular cells of the median eminence control blood flow to the ARC and subsequent ghrelin movement into the hypothalamus (Romanò et al., 2023). These cells are further regulated by pericytes to alter the permeability of fenestrated capillaries in this area. Evidence from studies in rats suggest that chronic stress results in an increase in pericyte numbers In the hippocampus, an increase associated with hyperpermeability in this region (Treccani et al., 2021). Therefore, the pericytes that surround the endothelial cells of CVOs, such as the median eminence, may underlie the stress-induced increases in Cy5-ghrelin labeling throughout the hypothalamus. Additional research, however, is required to determine if pericytes in the median eminence/ARC junction as well as in other CVOs are affected by social defeat stress.

The median eminence and third ventricle also contain tanycytes, a subset of glial cells that transport ghrelin into the blood–CSF system to be transported to other regions (Uriarte et al., 2019). There is also evidence that tanycytes participate in the transport of metabolic peptides, specifically leptin, into the brain (Balland et al., 2014); we did not examine if tanycytes were disrupted by chronic social defeat. There is evidence that obesity associated with chronic exposure to a high-fat diet or chronic glucocorticoid exposure changes the morphology of these cells and their expression of genes associated with negative energy states like deiodinase Type II (*Dio2*; Thaler et al., 2012; Wray et al., 2019). This needs to be examined in more detail, but there is a report suggesting that tanycytes don't express the GHSR (Uriarte et al., 2021), so it is possible that if these cells are modified by chronic social defeat, this is through stress-related signals other than ghrelin.

Ghrelin secretion is increased following a social defeat, suggesting that the X/A enteroendocrine cells that produce ghrelin may also display increased expression of the gene encoding for ghrelin. It is not known, however, if social defeat promotes the production of obestatin and desacyl-ghrelin, peptides also derived from the ghrelin gene. One could assume that the increase in circulating active ghrelin that is observed following chronic social defeat would also result in increased secretion of these two peptides, both of which seem to have feeding effects that oppose those of ghrelin (J. V. Zhang et al., 2005; Stengel et al., 2010). The mechanism of action of these two peptides remains elusive and does not seem to involve the modulation of GHSR signaling (Rhea et al., 2018). Thus, the role of these two peptides in modulating metabolic responses to social defeat stress requires further investigation.

It is important to highlight that this work was only conducted on male mice. The chronic social defeat paradigm used here relies on the natural behavioral responses associated with male territorial aggression and, therefore, cannot be easily applied in the same manner to female rodents. This is, inherently, a limitation of this paradigm, and therefore, our results may only be applicable to male mice. Recent adaptations to this model of social defeat have been developed to promote the inclusion of female rodents and some of these have been used to study blood–brain barrier changes in females. For instance, Dion-Albert et al. (2022) studied the effect of chronic stress in female rodents using a modified paradigm in which urine from male mice was applied to females before the introduction of the aggressive male counterpart. Results from their research show that the females displayed reductions in expression of the tight-junction protein, Cldn5, as previously demonstrated to occur in the NAc male mice (Menard et al., 2017). Notably, however, this reduction occurred within the prefrontal cortex of females and not males, suggesting that there may be sex differences in the location of blood–brain barrier leakiness (Dion-Albert et al., 2022). We are currently working on a modified social defeat paradigm that includes males and females and that results in sex differences in GHSR expression, suggesting a potential in sex differences in the ghrelin responses to social defeat in mice (Smith et al., 2023). Future work will investigate if chronic stress exposure also impacts ghrelin permeability in female mice.

Overall, changes in ghrelin entry into the brain of stressed mice suggest that chronic stress induces changes to neurovascular cells and that this allows for enhanced orexigenic effects of ghrelin. Increased ghrelin secretion and GHSR expression in response to chronic social defeat suggest that peripherally produced ghrelin can penetrate CNS regions beyond the circumventricular organs and have widespread effects, potentially associated with coping responses like eating. Thus, our results provide a rationale for increased ghrelin sensitivity and increased food intake in the face of stress, one that could be mediated by plasticity in the astrocytes surrounding the neurovascular cells of the blood–brain barrier.

## Abbreviations

ARC, arcuate nucleus; CNS, central nervous system; CVO, circumventricular organ; DMH, dorsomedial hypothalamus; GFAP, glial fibrillary acidic protein; GHSR, growth hormone secretagogue receptor; LH, lateral hypothalamus; NAc, nucleus accumbens; NTS, nucleus of the solitary tract; VMH, ventromedial hypothalamus; VTA, ventral tegmental area.

## Synthesis

Reviewing Editor: Tod E Kippin, University of California Berkeley Decisions are customarily a result of the Reviewing Editor and the peer reviewers coming together and discussing their recommendations until a consensus is reached. When revisions are invited, a fact-based synthesis statement explaining their decision and outlining what is needed to prepare a revision will be listed below. The following reviewer(s) agreed to reveal their identity: Mario Perello.

I have received two independent reviews of your manuscript. Although both reviewers felt the paper has significant merit and potential to advance the field, they also expressed concerns regarding the scope (particularly inclusion of only males), methodology and with some interpretations of the data presented in the manuscript. However, these concerns appear to be addressable but may require additional experimentation and analyses thus I encourage you to resubmit a revised version of your study. Below are the reviewers' comments, please address each comment in a revised version of the manuscript.

Reviewer#1

This is a very interesting set of experiments showing how chronic social defeat stress increases brain permeability to ghrelin in male mice. I have only a few 'big picture' comments.

1. Why male mice only? The authors should expand their experiments to female mice, or otherwise provide a strong rationale for limiting the work to males only.

2. Please provide the rationale for the N's chosen per each experiment (some experiments in particular seem to have a quite small N).

3. Would the authors speculate that these results are specific to acyl-ghrelin only or they may be extended to des-acyl-ghrelin as well? Unlike my comment below n. 1 (for which I feel strongly the need for expanding the experiments to female mice), here I don't feel necessarily that the authors should run new experiments with des-acyl-ghrelin, unless they wish to do so. However, the Discussion could include a brief comment on whether these results could or couldn't expand to des-acyl-ghrelin

Reviewer#2

The article entitled "Chronic Social Defeat Stress Increases Brain Permeability to Ghrelin in Male Mice" investigates whether social stress facilitates the entry of ghrelin into the mouse brain. First, the study reports that male mice exposed to social defeat stress for 21 days and systemically injected with Cy5-labelled ghrelin exhibit higher Cy5-ghrelin fluorescence in the hypothalamus, hippocampus, and midbrain. Furthermore, Cy5-ghrelin injections induce higher c-Fos expression in regions associated with the reward system in chronically stressed mice. Finally, the study shows that hypothalamic astrocytes in the ARC-median eminence junction display a reduction in their branching, suggesting increased blood-brain barrier permeability. Thus, the authors conclude that chronic stress results in increased accessibility of plasma ghrelin into the brain.

Overall, I think the study sheds light on an intriguing and paradoxical observation in the neuroendocrinology field: why the receptor for ghrelin is present in brain areas that do not seem to be targeted by plasma ghrelin in normal conditions. The experiments seem to be accurately designed. Data are clearly explained and seem to support the conclusions of the study. However, I found some issues that have drawn my attention, and I think authors should address them to improve the manuscript:

-The study examined and quantified differences in Cy5-ghrelin fluorescent signal throughout different regions of the mouse brain but does not provide any information regarding the choroid plexus, where fluorescent-ghrelin has also been shown to access after systemic treatment. Is Cy5-ghrelin fluorescent intensity in the choroid plexus affected in chronically stressed mice compared to non-stressed controls?

-The study shows that Cy5-ghrelin induced a significant increase in c-Fos expressing cells in stressed mice compared to non-stress counterparts. I would think that stress itself is able to increase the number of c-Fos expressing cells in some brain areas of stressed mice. Thus, I wonder why the vehicle-treated stressed mice have not been included in the neuroanatomical analysis.

-To assess the integrity of the blood vessels in the ARH, the authors used fluorescently tagged dextran and found no observable dextran signal in the non-stressed control mice but observed dextran accumulation in blood vessels in the ARC of stressed mice (Figure 5F). I think that these results would be more convincing by adding low magnification images of the regions of interest and providing a quantitative analysis of the fluorescent signal.

-In the discussion section, the authors argue that "Chronic social defeat increases NPY and AGRP mRNA expression in the ARC in a GHSR-dependent manner (Patterson et al., 2013), but does not increase GHSR mRNA expression in male mice (Smith et al., 2023)." Since the review process for eNeuro is double-blind for authors and reviewers, it is uncertain to me if the authors of the current manuscript have tested Ghsr mRNA levels in mice exposed to social stress. The assessment of GHSR in the brain of chronically stressed mice is important to rule out that the higher Cy5-ghrelin fluorescence labeling in the brain of chronically stressed mice is not due to an increase in the level of binding sites.

Others:

-The wording in lines 5-to-7 of the abstract should be fixed, as it seems to indicate that the ARH is a CVO, which is not the case, as well described in the introduction section of the manuscript.

-I think that prior work is properly cited, with the exception of two articles that should also be mentioned: DOI: 10.1159/000526245 and DOI: 10.1172/jci.insight.165763.

-Lines 330-331. The sentence "Importantly, we found that this effect was associated with increased expression of the peripherally injected Cy5-labelled ghrelin in the median eminence and ARC" does not sound right.

## Author Response

CSDS and Ghrelin Entry Reviewer Comments &Replies Reviewer#1 This is a very interesting set of experiments showing how chronic social defeat stress increases brain permeability to ghrelin in male mice. I have only a few 'big picture' comments.

1. Why male mice only? The authors should expand their experiments to female mice, or otherwise provide a strong rationale for limiting the work to males only.

RESPONSE: We appreciate the reviewer's concern regarding the exclusion of females from this experiment and agree that the investigation of female subjects is an important area that deserves investigating. Here, we did not include females in this experiment primarily due to constraints imposed by the chronic stress paradigm used. This experiment used the traditional model of social defeat stress, which relies on the innate territorial behaviors that male rodents display towards other males, and not towards females. Similarly, females do not display aggressive or territorial behaviors towards other females, make it difficult to employ social stressors that are relevant to both males and females. Our lab is committed to the inclusion of female rodents into our research on stress and metabolism and has been working diligently to test novel variations of social defeat stress that can be applied to females and do plan to expand this research project to investigate the blood-brain barrier in females. However, we cannot do so without significantly altering the entire stress paradigm and methodological approach that we used here. For context, this model of social defeat was selected based on the criteria that 1) it has been well demonstrated that this stress paradigm results in elevated ghrelin secretion (DOI: 10.1038/nn.2139 and 10.1210/en.2012-1834) and 2) changes to blood-brain barrier permeability that have been demonstrated using this paradigm (DOI: 10.1038/s41593-017-0010-3). We do plan to investigate changes to ghrelin entry and associated blood brain barrier changes in females, and are currently validating social defeat stress paradigms where females can be included. Validating models along with doing a duplicate of the experiments to include females would be impossible to do especially with a time limit of three months to resubmit the paper. In fact, the project as presented is the result of about 2 years of work, so we have address this limitation in the discussion. Thus, while we are unable to conduct the suggested additional experiments for this publication, we have added these limitations and future directions to our discussion (see page 22) to highlight the importance of the inclusion of females. We thank the reviewer for this important critique and hope that this adequately addresses their concerns.

2. Please provide the rationale for the N's chosen per each experiment (some experiments in particular seem to have a quite small N).

RESPONSE: We appreciate the reviewer's attention to the sample sizes in our experiments and we agree that ensuring adequate sample sizes is fundamental to the validity of our results.

Regarding the quantification of astrocyte morphology, we determined that a sample size of 5/group would be adequate as we measured 6 cells/animal/group, resulting in the quantification of 30 cells/group. Quantification of multiple cells per animal has been demonstrated to be an appropriate method to adequately power the analyses and has been reported in publications employing similar methods (DOI: 10.1093/ijnp/pyab052). Additionally, we provided the η2 values corresponding to our analyses to illustrate that the effect size reported was not impacted by the sample size.

We recognize that our sample size for the dextran analysis was smaller than ideal, however, we believed that a general analysis of overall blood-brain barrier permeability/leakiness would strengthen the credibility of our findings. Our small sample size was partially due to challenges associated with intravenous retroorbital injections and time limitations restricting us from conducting additional experiments. In an attempt to mitigate the impact of this limitation we have quantified the dextran signal from n=6 arc sections/animal, resulting in the analysis of 24 sections per group. This produced a significant result (p=0.003), with a large η2 value (η2 =0.79), thus highlighting that our result was not limited by the sample size.

Despite these limitations we believe that the level of significance obtained and the effect size suggestthat the data are robust and provide valuable results pertaining to cellular changes in the blood-brain barrier.

3. Would the authors speculate that these results are specific to acyl-ghrelin only or they may be extended to des-acyl-ghrelin as well? Unlike my comment below n. 1 (for which I feel strongly the need for expanding the experiments to female mice), here I don't feel necessarily that the authors should run new experiments with des-acyl-ghrelin, unless they wish to do so. However, the Discussion could include a brief comment on whether these results could or couldn't expand to des-acyl-ghrelin RESPONSE: We have added the potential for des-acyl ghrelin to this to the discussion section (see poage 21). The role of des-acyl gfhrelin (and obestatin) on stress remains elusive. We have not attempted to measure des-acyl ghrelin or obestatin simply because most of the plasma we collect is used for CORT and ghrelin assays and it is difficult to save enough of the sample for additional assays. Also, the literature on the role of these peptides on feeding would be difficult to link to the data on receptor sites given that these peptides seem to have a biological effect that is not mediated by the GHSR. The field in general needs tio answer some very basic questions about these peptides before leaping to their role on stress, but definitely something that is in our radar.

Reviewer#2 The article entitled "Chronic Social Defeat Stress Increases Brain Permeability to Ghrelin in Male Mice" investigates whether social stress facilitates the entry of ghrelin into the mouse brain. First, the study reports that male mice exposed to social defeat stress for 21 days and systemically injected with Cy5-labelled ghrelin exhibit higher Cy5-ghrelin fluorescence in the hypothalamus, hippocampus, and midbrain. Furthermore, Cy5-ghrelin injections induce higher c-Fos expression in regions associated with the reward system in chronically stressed mice. Finally, the study shows that hypothalamic astrocytes in the ARC-median eminence junction display a reduction in their branching, suggesting increased blood-brain barrier permeability. Thus, the authors conclude that chronic stress results in increased accessibility of plasma ghrelin into the brain.

Overall, I think the study sheds light on an intriguing and paradoxical observation in the neuroendocrinology field: why the receptor for ghrelin is present in brain areas that do not seem to be targeted by plasma ghrelin in normal conditions. The experiments seem to be accurately designed. Data are clearly explained and seem to support the conclusions of the study. However, I found some issues that have drawn my attention, and I think authors should address them to improve the manuscript:

-The study examined and quantified differences in Cy5-ghrelin fluorescent signal throughout different regions of the mouse brain but does not provide any information regarding the choroid plexus, where fluorescent-ghrelin has also been shown to access after systemic treatment. Is Cy5-ghrelin fluorescent intensity in the choroid plexus affected in chronically stressed mice compared to non-stressed controls? RESPONSE: Following this suggestion, we revisited our samples and attempted to analyze the Cy5-ghrelin signal observed in other circumventricular regions like the choroid plexus, OVLT and SFO. Unfortunately, due to difficulties in maintaining the structure of the choroid plexus when processing the brain, we did not have a large number of samples that contained these regions. We were, therefore, unable to quantify these results, however, as displayed in the corrected figure 1, the Cy5-ghrelin signal did appear brighter in the stressed mice. We have added a short description of these results, amended our methods to include that we looked at this, and discussion the results adding the need for further investigation. We also looked for the area postrema but we did not have sections that were cut that far back from the brains in this study.

-The study shows that Cy5-ghrelin induced a significant increase in c-Fos expressing cells in stressed mice compared to non-stress counterparts. I would think that stress itself is able to increase the number of c-Fos expressing cells in some brain areas of stressed mice. Thus, I wonder why the vehicle-treated stressed mice have not been included in the neuroanatomical analysis.

RESPONSE: We did analyze the cFOS counts from both the non-stress and stress vehicle injected mice and determined that, in the regions displayed, there were no significant differences. It important to note that c-fos data were collected from mice that were injected 24hrs. after their last stressor, so the effect of the stressor on c-Fos are not evident if the animals were injected with saline. Therefore, we pooled the vehicle-injected group to include both the non-stress and stressed mice, allowing us to emphasize the effect of the ghrelin-injection on neuronal activation following stress exposure. To increase the transparency of the data, we have added an additional label to the graphs presented in figure 4 to clearly indicate that the vehicle injected group contained both non-stress and stressed animals.

-To assess the integrity of the blood vessels in the ARH, the authors used fluorescently tagged dextran and found no observable dextran signal in the non-stressed control mice but observed dextran accumulation in blood vessels in the ARC of stressed mice (Figure 5F). I think that these results would be more convincing by adding low magnification images of the regions of interest and providing a quantitative analysis of the fluorescent signal.

RESPONSE: We thank the reviewer for this comment and we have addressed it by conducting additional analyses enhance the validity of our result. Figure 5 has been edited to include a high magnification image of the median eminence and ARC that clearly demonstrates dextran accumulation in this junction of the stressed mice. Additionally, quantification of the dextran signal in the ARC has been added which illustrates a significant increase in dextran fluorescence in the ME/ARC junction in the stressed animals.

-In the discussion section, the authors argue that "Chronic social defeat increases NPY and AGRP mRNA expression in the ARC in a GHSR-dependent manner (Patterson et al., 2013), but does not increase GHSR mRNA expression in male mice (Smith et al., 2023)." Since the review process for eNeuro is double-blind for authors and reviewers, it is uncertain to me if the authors of the current manuscript have tested Ghsr mRNA levels in mice exposed to social stress. The assessment of GHSR in the brain of chronically stressed mice is important to rule out that the higher Cy5-ghrelin fluorescence labeling in the brain of chronically stressed mice is not due to an increase in the level of binding sites.

RESPONSE: Revisions have been made to the discussion section to emphasize our results. We clarified that there are no changes in GHSR mRNA expression in the ARC of male mice following chronic social defeat, however, recently published data has illustrated that GHSR expression is increased in the VTA of male mice following social defeat. We believe that these findings strengthen our data that demonstrate that ghrelin movement into the brain occurs at the median eminence and ARC junction, while increased movement and activation of non-hypothalamic brain regions is important in mediating stress-induced feeding. We hope that this clarifies our results and strengthens the conclusions of this research.

Others:

-The wording in lines 5-to-7 of the abstract should be fixed, as it seems to indicate that the ARH is a CVO, which is not the case, as well described in the introduction section of the manuscript.

RESPONSE: Revised to read "The ability of ghrelin to enter the brain, however, seems to be restricted to circumventricular organs like the median eminence and the brain stem area postrema (AP), whereas ghrelin does not readily enter other GHSR expressing regions like the ventral tegmental area (VTA)." -I think that prior work is properly cited, with the exception of two articles that should also be mentioned: DOI: 10.1159/000526245 and DOI: 10.1172/jci.insight.165763.

RESPONSE: We have added the results from these studies to our discussion section.

-Lines 330-331. The sentence "Importantly, we found that this effect was associated with increased expression of the peripherally injected Cy5-labelled ghrelin in the median eminence and ARC" does not sound right.

RESPONSE: Revised to read "Importantly, we found that this effect was associated with an increase in Cy5-labelled ghrelin in the median eminence and ARC."
